# Supplementary material for: Patterns of Intron Gain and Loss in Fungi
Source: PLoS Biol. 2004 Nov 30;2(12):e422. doi: 10.1371/journal.pbio.0020422 (PMC532390; doi:10.1371/journal.pbio.0020422)
Supplement: Table S1 — Also available at http://genes.mit.edu/NielsenEtAl/. (4.3 MB ZIP). [file pbio.0020422.st001.zip › NielsenEtAl/html/771.html]

AN2128.1.NCU06277.1.MG05272.1.FG02553.1


```
 CLUSTAL W (1.82) Multiple Sequence Alignments - Introns Inserted


Sequence 1: MG05272.1	784 aa
Sequence 2: FG02553.1	767 aa
Sequence 3: NCU06277.1	785 aa
Sequence 4: AN2128.1	774 aa
Alignment Length: 840 aa
Number Identitical Residues: 297 aa
Alignment Score (without introns) 14980


MG05272.1 	--MDASYLSQQVNTIIDQLHGLFDEIGVPSHERESRETE0LFSALSDTLQKQVRSVNS~E
NCU06277.1	--MDTSYLAQQVNTIVGQLHGLFDEIGVAHNDREHREAE0N----------QVRRVTN~E
FG02553.1 	-MVDTSYLAQQVNNSIGQLHGLFDEIGVPDHEREARESE0LFSALSEALNSQVRLVNS~E
AN2128.1  	MGVDTSYLTTQVNNIVSQLHGIFDEIGVPSHERESREAE0LFSALSETLNNHLKLVDD2E
          	  :*:***: ***. :.****:******. ::** **:*   :: :.: ..::: * . *

MG05272.1 	KKAMVEEAQRIITIIRQMEASLDDSQSHRDY-SSGDDLKITYPLTRCLQGLKEKHQQISK
NCU06277.1	KQEMIEEAQGIITTIRQMEAAMDDTKKARRS-LEDDGLTVTYPLVQCIQSLQEKHSQVAR
FG02553.1 	KKEMVDEAKKIITIIHQMEASLDD-QNRRRS-NEDDELTITYPLTRCLQKLKEKHTIISR
AN2128.1  	KNAMTEEAHKLINAIQQMEESLVDEKANGQYSLDNDDLQVTYPLNRCVAFLREQHSAIGK
          	*: * :**: :*. *:*** :: * :     : ..* * :**** :*:  *:*:*  :.:

MG05272.1 	LHKDRFEQVKK1LVVALESYSSHLESSFVKLRLPPTGPNQSIPPDFDLSPSYVQKLDDEF
NCU06277.1	AHRERYEEIKK1LAQALESYSLHLEPGFVKLELPPTGPDQPVSTTFDLTDSYVQKLDEEF
FG02553.1 	IHKERFEQVKK1LVVALESYSSHLEPTFVQIPLPPTDPDQTIPANFDLSSTYVDKLDSEF
AN2128.1  	LHRQRFEQVKK1LVEALESYSSHLEPSFVTLELPPTAPGSSVAPSFDLSPTYVTALDNEF
          	 *::*:*::** *. ****** ***. ** : **** *...:.. ***: :**  **.**

MG05272.1 	TRVYEEYTRRVTTVKAICEHIISLWAELGTPQAQTDGAIVKYYRDAPEQLGLHEEDIAQL
NCU06277.1	TRVFEEYTRRVAAVKSVSEDIIQLWAELGTPQAQTDGAIVKYYRDAPEQLGLHQVDIERL
FG02553.1 	TRVYEEYSRRIATVQALADQTIGLWAELGIPQAQQDAAIVKYYRDAPEQLGLHEEDISRL
AN2128.1  	TRVYEEYHRRLELVTTTCEEIIKLWAELGTPQAQTDSSIVQHYRESPEQLGLHESDLANL
          	***:*** **:  * : .:. * ****** **** *.:**::**::*******: *: .*

MG05272.1 	RSKRDKLAEEKKSREKRLRDLKAAVEALWEKLGIEEHERKKFLNANRGCGVRQINEFEDE
NCU06277.1	RAKKDKLADEKKNRENRLANLKATVGELWEKLGVDESVRKAFLNSNRGCGLRQIHEFEDE
FG02553.1 	RAKRDRLTDEKKNREKKLNQLRAAVEALWIKLGVDESETKSFLNANRGCGVRQINEFEDE
AN2128.1  	LARREKLLDEKRGRERKLKDLKHAVEGLWERFGVEECDRKAFLAANRGCGLRTINEFEEE
          	 :::::* :**:.**.:* :*: :*  ** ::*::*   * ** :*****:* *:***:*

MG05272.1 	LGRLNELKRQNLHLFVEDARFKLQELWDALYLCEDEMLEFTPAFSDVYSDALLEAHEREI
NCU06277.1	LGRMNELKRQNMHIFIEDARVKLQGLWDALYYSEDEMLEFTPAFSDVYSDALLEAHEREI
FG02553.1 	LSRLNELKRQNLHLFVEDTRVKLQELWDALYFSEDEMLEFTPAFSDVYSDALLEAHEREV
AN2128.1  	LSRLNELKRQNLHLFVEDARCRLQELWDGLYYSEEEMLDFTPAFSDVYSDALLEAHEAEI
          	*.*:*******:*:*:**:* :** ***.** .*:***:****************** *:

MG05272.1 	SRLEALKEQRAPILALVERHRSLVTERDDLAASSQDASRLMMKGQKGERRDPGKLLREEK
NCU06277.1	ARLETLKEQRAPLLSLVDKYKSLVADREELAASSQDASRLLMKGQKGERRDPGKLLREEK
FG02553.1 	ARLEALREERAPILALIDRRKALIKERNDLAASSQDASRLMGRGQKGEKRDPGKLLREEK
AN2128.1  	ARLEALKEQRAPILNLIDKHRSLLAEREALAVSSQDASRLMARGNKGERRDPGKLLREEK
          	:***:*:*:***:* *::: ::*: :*: **.********: :*:***:***********

MG05272.1 	MRKRIAKELPKITVELRKGLEKWEDEWGRDFLVHGERYLDVIEASES--KPAVPP-RSKT
NCU06277.1	MRKRIAKELPKVTVDLRKALEQWEGEYDRPFLVHGERFLDSVEEEDP--KTGLGTSRPKT
FG02553.1 	MRKRIAKELPKITAELRRILSDWEEEYSRPFLVCGECYLDEIEAEEDSKKNALPP-RSKT
AN2128.1  	MRKRIAKELPKVETDLRKELENFEDEYGRPFLVQGERYLDELTKVAA-----KPPARSKT
          	***********: .:**: *..:* *:.* *** ** :** :            .:*.**

MG05272.1 	PGAGAVRTAVSMAGPAATTRPGTAMKSNAPPSRTNSSATRPTTARPPSQHATAKTPTTAT
NCU06277.1	P------------GVS----ASAAKPRERAATLSRADSAHALRGQPP--RSPTKTPSAST
FG02553.1 	P-----------AGPP----PSAAKPSKSVPSRIN------TKSIPP--RSMTKTPTTAG
AN2128.1  	P------------GPS--STTKRNAPPARPASAMNRPGSSMRGPPPP--RSTTKTPTSQP
          	*            * .  : .         .:  .  .:      **  :: :***::  

MG05272.1 	SGTGTLRRAPQPTAGHTTTSKGSPSRIPARAPLSNLKHGNNSPER---PRPESRGD--AF
NCU06277.1	PSLPTRSATINGKS-------GSPTRLPARAPLSNLTFINSAPVDRITGRPESRVDGTGT
FG02553.1 	PPKRPGHQTHVSTSSIPLSPVRSPSRIPARVPLSNMKYGNNSPER---PRAESCAD--NY
AN2128.1  	TKYNTVGASRSGAK--------SPTKIPARVPLSNMPHGNNSADR----RGPSTYSSSTV
          	.   .   :             **:::***.****: . *.:.      *  *  ..:  

MG05272.1 	RNAPPLMRAPPPKMRDLVPPPEARLETPPSPYRREDFGMSS-NVRQVETDDIYDDRNERS
NCU06277.1	LRGAPLLRAPPPKMRSLLP--PPDLEKPNNPYNGANLRSSITLIREVEAEDMYDDRG-SS
FG02553.1 	RPGQSMMRAPPPKMRDIMA--QAEFEHNMNPYKSSNLACS--IVRPVEPEDVYDDRPPTA
AN2128.1  	NGKLPSSRAPPPRMRALTTTDKEERGSYLFEPPRCASAMSNSFVRPVSPEDVYDDRQRSF
          	    .  *****:** : ..                   * : :* *..:*:****    

MG05272.1 	R----QLQARTKSHQDLRGSHESYESHTSRDKAYA-QSTYAHATYSHPP---RQISN---
NCU06277.1	R------SSTRPGHSNSISSHTSRTSHSSLTSYTASQSSFKHLPQAPPPPV-RQVSDRE-
FG02553.1 	RSNSNHSQYSYPSNSSHLSSHPSFSEESFQDPYAT-IRSQGYRPAAPPP---RHISGES-
AN2128.1  	MSSSIFSQRSTGFSQSSQSSASSVSLKSSLQGQFP--RPNPYLQHAPPPPAPRQVSNASS
          	 :.:   .      ..  .*  *   .:      .   .  :   : **. .*::*. .:

MG05272.1 	---TSTAVSGSENWETYDD-NSEPEADASDSYYEKMRAARGK----RMTPEMGYARPHAS
NCU06277.1	--SAGSAVSESENWQTYED-GSDLEEEAGNLYLHKVRGARSLGGFRRSTPDDSRPQSHAS
FG02553.1 	--MASTNISGSENWETYDDDNSEPEVDVSDAYYARLRATRSK----QFSPDQG--RPISS
AN2128.1  	ASAADTITTGSENWETFDD-GSESDGDASEAYYARLRAAHGK----RLAPEDQQPSISLA
          	:: :.:  : ****:*::* .*: : :..: *  ::*.::.     : :*:   .    :

MG05272.1 	Q------------------AKRPRGLPPG-AHPSLAMVDPEGTRIVSG-SEWTDE-DGF-
NCU06277.1	QHSQSQLGFSQLQQAAQLRAMKQSGLQPPAHAGRVALVDADGDRIMGGQNEWADE-DGYS
FG02553.1 	Q------------------SKRIRGVPPAGSYNGPVMIDQDGNRIISG-SEWTDE-DAY-
AN2128.1  	G-------------------KKAKGIRSVSPDEP---IGRHMVRVAGSDNGWTDDMEAY-
          	                     :  *: . .       :. .  *: .... *:*: :.: 

MG05272.1 	---
NCU06277.1	HYH
FG02553.1 	---
AN2128.1  	---
          	
```
